# Tetrodotoxin, a Potential Drug for Neuropathic and Cancer Pain Relief?

**DOI:** 10.3390/toxins13070483

**Published:** 2021-07-12

**Authors:** Rafael González-Cano, M. Carmen Ruiz-Cantero, Miriam Santos-Caballero, Carlos Gómez-Navas, Miguel Á. Tejada, Francisco R. Nieto

**Affiliations:** 1 Department of Pharmacology, and Neurosciences Institute (Biomedical Research Center), University of Granada, 18016 Granada, Spain; rgcano@ugr.es (R.G.-C.); rcmaric@ugr.es (M.C.R.-C.); msantosc@ugr.es (M.S.-C.); carlosgna98@correo.ugr.es (C.G.-N.); 2 Biosanitary Research Institute ibs.GRANADA, 18012 Granada, Spain; 3 INCLIVA Health Research Institute, 46010 Valencia, Spain; matejada@incliva.es

**Keywords:** tetrodotoxin, TTX, voltage-gated sodium channels, neuropathic pain, cancer pain

## Abstract

Tetrodotoxin (TTX) is a potent neurotoxin found mainly in puffer fish and other marine and terrestrial animals. TTX blocks voltage-gated sodium channels (VGSCs) which are typically classified as TTX-sensitive or TTX-resistant channels. VGSCs play a key role in pain signaling and some TTX-sensitive VGSCs are highly expressed by adult primary sensory neurons. During pathological pain conditions, such as neuropathic pain, upregulation of some TTX-sensitive VGSCs, including the massive re-expression of the embryonic VGSC subtype Na_V_1.3 in adult primary sensory neurons, contribute to painful hypersensitization. In addition, people with loss-of-function mutations in the VGSC subtype Na_V_1.7 present congenital insensitive to pain. TTX displays a prominent analgesic effect in several models of neuropathic pain in rodents. According to this promising preclinical evidence, TTX is currently under clinical development for chemo-therapy-induced neuropathic pain and cancer-related pain. This review focuses primarily on the preclinical and clinical evidence that support a potential analgesic role for TTX in these pain states. In addition, we also analyze the main toxic effects that this neurotoxin produces when it is administered at therapeutic doses, and the therapeutic potential to alleviate neuropathic pain of other natural toxins that selectively block TTX-sensitive VGSCs.

## 1. Introduction

Chronic pain is a serious public health and socioeconomic problem that affects millions of people worldwide each year [1]. Some chronic pain conditions can be a consequence of abnormal functioning of the nervous system. In particular, neuropathic pain arises from a lesion or disease of the somatosensory system at peripheral or central level [2]. Peripheral neuropathic pain is the result of injuries to the peripheral nervous system induced by various conditions, including metabolic diseases, infections, mechanical trauma, and chemotherapy [3]. Central neuropathic pain involves injuries to the central nervous system, commonly caused by spinal cord injury, stroke, and multiple sclerosis [4]. The initial lesions involved in both forms of neuropathic pain trigger a cascade of changes that lead to maladaptive plasticity within the nervous system and inevitably to neuropathic hypersensitivity [5]. The prevalence of chronic pain with neuropathic characteristics has been estimated to be in the range of 7–10% [6] and it is expected to rise in the future [3].

Cancer-related pain is a very invalidating symptom, affecting approximately 66% of patients who suffer from cancer [7]. Cancer-related pain frequently presents a mixed pathophysiology, including both nociceptive and neuropathic components, depending on the progression and extension of tumor, being often very difficult to differentiate between acute and chronic pain [8].

Commonly used analgesics for nociceptive pain, such as non-steroidal anti-inflammatory drugs (NSAIDs) and opioids, are less effective in neuropathic [9] or cancer pain [10]. In fact, among cancer patients, those with cancer-related neuropathic pain usually have more trouble to optimally control pain [11]. First-line drug therapies for neuropathic pain include tricyclic antidepressants (TCAs), serotonin-norepinephrine reuptake inhibitors (SNRIs), gabapentinoids, and topical application of lidocaine or capsaicin [12]. These medications can be effective, but carry the potential risk of many side effects [12]. Opioids are lower-line drug options for neuropathic pain, as there is low evidence to support the use of opioids for long-term in chronic neuropathic pain, with possible benefit only for short-term use [9,13]. Therefore, despite the multiple agents available for the treatment of neuropathic pain, current treatment options provide an average reduction of neuropathic pain by 30–50% [9]. The effectiveness of available drugs in the treatment of cancer pain is also very low [14]. The difficulty in optimally treating these pain conditions has prompted significant research into new analgesic agents with fewer side effects.

Tetrodotoxin (TTX) is a potent non-peptide guanidinium neurotoxin that blocks voltage-gated sodium channels (VGSCs) found in puffer fish and other marine and terrestrial animals [15]. VGSCs play an important role in pain and, in particular, numerous studies have shown that some of the TTX-sensitive VGSC subtypes are strongly implicated in the pathophysiology of chronic pain, especially neuropathic pain [16]. Since TTX blocks this subset of VGSCs in a highly selective manner, this agent may have a potential role in relieving neuropathic pain. In fact, this agent is progressing in its preclinical and clinical development, having been evaluated in various clinical trials for the indications of cancer-related pain (NCT00725114, NCT00726011) and chemotherapy-induced neuropathic pain (NCT01655823). 

The main objective of this review is to examine the available evidence from preclinical and clinical studies conducted to elucidate the therapeutic potential of TTX to alleviate neuropathic pain, as well as pain associated with cancer. In addition, we also analyze the main toxic effects that this neurotoxin produces when it is administered at therapeutic doses. Finally, we describe the therapeutic potential to alleviate neuropathic pain that has been described for other natural toxins that selectively block TTX-sensitive VGSCs.

## 2. Role of TTX-Sensitive Voltage-Gated Sodium Channels (VGSCs) in Neuropathic and Cancer Pain

**Figure 1:**
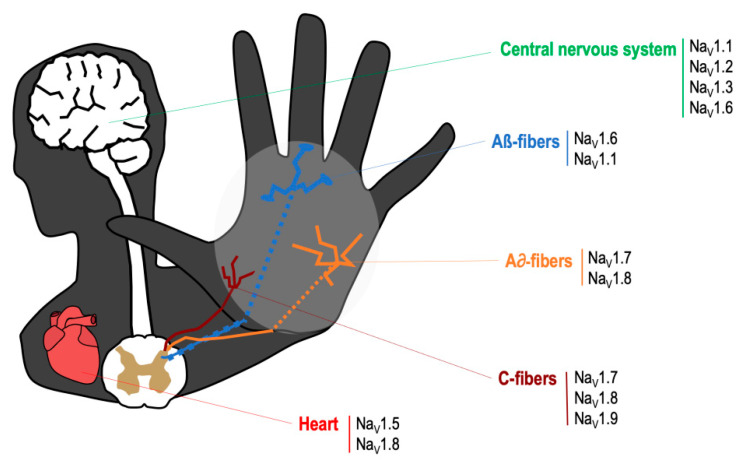
Main location of voltage specific sodium channels in the nervous system.

The VGSCs are integral membrane proteins composed of a 260 kDa α-subunit and one or more β-subunits, being the α-subunit responsible for forming the pore and for the main biophysical properties of the channel [16]. Most VGSCs are located at peripheral and/or central nervous system (see Figure 1), and are responsible for generating the Na^+^ currents that lead to the initiation and propagation of neuronal action potentials [17]. TTX binds to VGSCs by the interaction between the positively charged guanidine group in the TTX molecule with the negatively charged carboxylate residues that are placed around the outer vestibule of the channel; this binding occludes the channel pore and blocks Na^+^ current [18]. VGSCs are classified according to its sensitivity to TTX. Thus, TTX-sensitive VGSCs (Na_V_1.1, Na_V_1.2, Na_V_1.3, Na_V_1.4, Na_V_1.6 and Na_V_1.7) are blocked by nanomolar concentrations of this neurotoxin, whereas TTX-resistant VGSCs (Na_V_1.5, Na_V_1.8, Na_V_1.9) are inhibited at micromolar concentrations [16]. The mechanism of action by which TTX exerts its analgesic effect is by binding to the α-subunit within the outer vestibule of the VGSC, blocking the entry of Na^+^ ions through the channel [16,18]. In this manner, TTX reduces the Na^+^ ionic fluxes required for the initiation and conduction of nerve impulses.

Nociceptive pain has a physiological protective role in preventing tissue injury. Under normal conditions, primary nociceptive neurons generate action potentials only when they are stimulated with a noxious stimulus [5]. Following nerve injury, nociceptive neurons can begin to discharge spontaneously in an abnormal way, and they reduce their response threshold and increase their discharge frequency, evoked by stimulation. These processes are responsible for inducing hyperexcitability of primary sensory neurons (peripheral sensitization) [5]. In addition, ongoing abnormal activity originating in injured peripheral nerves can be the trigger of central sensitization [19]. Peripheral and central mechanisms, occurring sequentially and concurrently, are responsible for the induction and maintenance of neuropathic pain [5].

VGSCs play a role in persistent and chronic pain as it has been reported that changes on VGSC expression in primary sensory neurons, their abnormal accumulation at the site of injury, as well as posttranslational modifications of these channels, occur after nerve injury, and lead to peripheral sensitization processes [20] (see Table 1). 

Thus, it has been shown a massive upregulation of the expression of the TTX-sensitive VGSC Na_V_1.3 in peripheral sensory neurons following nerve injury, in several models of neuropathic pain in rodents [21,23,24,26,33], as well as in patients [28,29]. Na_V_1.3 subtype might account for neuronal hyperresponsiveness during neuropathic pain as this isoform is not expressed in peripheral sensory neurons in normal condition (only during embryonic development) [20], but as mentioned before, it dramatically upregulates following nerve injury in animals and patients with neuropathic pain (see Table 1). However, specific reduction in the expression of Na_V_1.3 subtype alone (with intrathecal administration of antisense oligonucleotides or with knockout mice), is not sufficient to reduce behavioral hypersensitivity associated with nerve injury [46,47]. 

Another TTX-sensitive VGSC that has received much attention is Na_V_1.7 subtype. This VGSC is predominantly localized in the peripheral nervous system, and many studies have reported a strong correlation between Na_V_1.7 function and many pain states in rodents and humans [20]. However, the role of Na_V_1.7 in neuropathic pain remains uncertain, as contradictory results have been reported in rodents and humans, with some studies showing upregulation [27,34,40,41,45], and others downregulation [24,28,39] in primary sensory neurons. In addition, mice lacking Na_V_1.7 normally developed neuropathic pain after peripheral nerve injury [48]. Interestingly, it has been shown that Na_V_1.7 could have a more relevant role in the somatosensory system of humans than that of mice, since Na_V_1.7 is the predominant VGSC in human dorsal root ganglia (DRG) (50% of total VGSC expression), whereas in mice, this subtype represents only a small percentage (18% of total VGSC expression) [49]. In addition, in the same study, it is shown that human DRG neurons in primary cultures treated with paclitaxel (an antitumor drug which is known to induce neuropathic pain), increase the expression of Na_V_1.7 [49]. Finally, the importance of Na_V_1.7 for pain in humans is revealed by the existence of loss-of-function mutations of Na_V_1.7 gene (SCN9A) that lead to congenital insensitivity to pain, and gain-of-function mutations of this gene, which lead to congenital pain disorders characterized by hyperexcitability and extreme pain [50].

More recently, it has been shown that Na_V_1.6 also plays a relevant role in neuropathic pain of different etiologies, as several studies have reported that Na_V_1.6 is upregulated during neuropathic pain of different etiologies [35,36,37,38]. In addition, specific inhibition of Na_V_1.6 with small interfering RNA injected into the DRG [51], or knockout mice [52], reduce neuropathic pain in rodents. On the other hand, the involvement in neuropathic pain of other TTX-sensitive VGSCs present in the nervous system (Na_V_1.1 and Na_V_1.2) is not totally clear [16]. 

TTX-resistant VGSCs which are preferentially expressed in primary afferent neurons (Na_V_1.8 and Na_V_1.9) are involved in some pain conditions [20]. However, the role of these VGSC subtypes in neuropathic pain is also unclear since it has been described that they are downregulated in DRG after nerve injury [23,24,53,54]. In addition, experiments with knockout mice show a role of Na_V_1.9, and to a lesser extent or Na_V_1.8 in the generation of cold hypersensitivity, but not mechanical hypersensitivity in models of neuropathic pain [55]. On the other hand, the principal cardiac VGSC Na_V_1.5 is not involved in pain [17], but it is of particular interest, as any VGSC blocker with analgesic potential, should not inhibit Na_V_1.5 at therapeutic doses, for obvious safety reasons. Interestingly, Na_V_1.5 is considered a TTX-resistant VGSC [56].

In summary, the scientific literature shows that several subtypes of TTX-sensitive VGSCs (Na_V_1.3, Na_V_1.6 and Na_V_1.7) play a relevant role in the generation and maintenance of neuropathic pain. Therefore, selective pharmacological suppression of the abnormal activity of the different TTX-sensitive VGSCs contributing to neuropathic pain may represent a potential therapeutic tool for neuropathic pain relief. This is the rationale for the use of TTX in neuropathic chronic pain conditions.

## 3. Effects of TTX in Preclinical Models of Neuropathic and Cancer Pain

### 3.1. Preclinical Studies on Neuropathic Pain

TTX has been widely employed in vitro to characterize the role of VGSCs in nociception and chronic pain. In addition, the effects of TTX on pain behaviors have been studied in several models of acute and chronic pain. Thus, the systemic administration of TTX has shown analgesic effect in classical pain models such as in the writhing test in mice [57] and rats [58], and in the formalin test in rats [57]. In another study, systemic TTX showed a significant reduction of mechanical hyperalgesia in a rat model of inflammatory pain induced by intraplantar carrageenan [59]. Our group demonstrated that the systemic administration of TTX reduced pain behaviors in different models of visceral pain in mice [60].

As we have described in the previous section, some TTX-sensitive VGSCs play a key role in neuropathic pain, and this probably explains why the effect of TTX has been more widely tested in preclinical models of neuropathic pain (see Table 2).

Lyu and collaborators [62] reported the first study on TTX effects in a model of neuropathic pain in 2000. These authors showed that mechanical allodynia induced by spinal nerve ligation (SNL) was significantly attenuated by the topical application to the DRG of TTX at low doses that did not block action potential conduction. This study also reported that TTX ameliorates mechanical hypersensitivity when applied to the epidural space but not when this neurotoxin was administered intraperitoneally, suggesting a local effect of TTX. However, the dose of TTX used in this study (25 nM) was probably too low to achieve a systemic analgesic effect. In a later study was reported that the nerve blockade with TTX immediately following nerve injury, prevented the development of thermal and mechanical hypersensitivity, and the spontaneous afferent activity, in two rat models of neuropathic pain, the chronic constrictive injury (CCI) and the spared nerve injury (SNI) models [61]. However, when nerve blockade was performed once the neuropathic pain was established (10 days after nerve injury), it only transiently inhibited mechanical hypersensitivity in both neuropathic pain models, suggesting that the local inhibition of TTX-sensitive VGSCs during the initial stage of neuropathic pain might be sufficient to inhibit the subsequent development of neuropathic pain [61]. Another report showed that the preventive local application of TTX onto the median nerve inhibited the development of tactile allodynia subsequent to the chronic constriction injury of this nerve, in parallel with a reduction of the increase of astrocyte activation in the cuneate nucleus [63].

Two independent studies evaluated the effects of systemic administration of TTX in neuropathic pain models induced by surgical nerve damage. Marcil and collaborators [57] tested the ameliorative effects of several doses of subcutaneous TTX on pain-like behaviors induced by the partial sciatic nerve ligation (pSNL) model in rats and compared such effects with those produced by morphine. They found that TTX reduced mechanical allodynia and thermal hyperalgesia, without affecting the contralateral non-injured side. In contrast, the administration of morphine reduced neuropathic pain, but it also led to an increase in pain threshold strikingly above preoperative values in both ipsilateral and contralateral paws, although the dose of morphine was probably too high. In another report, it was shown that acute and subchronic subcutaneous treatment with TTX suppressed mechanical and thermal hyperalgesia and mechanical allodynia in the rat CCI model [67]. In the same study, the authors evaluated the effects of subcutaneous TTX on pain behaviors induced by the CCI of the infraorbital nerve in rats, and found that TTX had only modest analgesic effects in this pain condition. They also provided data about the possible mechanisms that contribute to the ameliorative effects of TTX in neuropathic pain. In particular, they showed that acute subcutaneous administration of TTX attenuated the increase of c-Fos expression (widely used as marker of neuronal activity) in the dorsal horn of the spinal cord and in some supraspinal areas in response to light mechanical stimulation of the ipsilateral hind paw. In addition, they found that TTX alleviated pain-related behaviors induced by nerve injury through mechanisms that might involve complex interactions with opioidergic systems, whereas noradrenergic and serotoninergic systems were not involved [67].

TTX was also evaluated in several models of cancer chemotherapy-induced neuropathic pain with contradictory results. Thus, the intraperitoneal administration of TTX did not attenuate mechanical allodynia induced by vincristine in rats, whereas other drugs including morphine, pregabalin, mexiletine and lidocaine did [64]. In a more recent study, Alvarez and Levine [70] showed that intramuscular TTX produced a small but significant reduction of neuropathic muscle pain induced by the antineoplastic agent oxaliplatin in rats. Our group found that acute TTX administered subcutaneously attenuated mechanical and cold allodynia and heat hyperalgesia induced by paclitaxel in mice, once the neuropathy was already established [65]. In addition, we also showed that the preventative administration of TTX completely prevented the development of mechanical and cold allodynia, without affecting heat hyperalgesia. Such findings open the possibility of preventative treatment of neuropathic pain induced by chemotherapy in patients, where the moment of onset of the nerve insult is known and preventive treatment could be possible. The mechanism by which TTX prevents chemotherapy-induced neuropathic pain is unknown. However, it has been shown that chemotherapy-induced neuropathy is associated with upregulation of some TTX-sensitive VGSCs in the DRG and in the spinal cord (see Table 2). It is possible to speculate that blockade of the early spontaneous afferent activity generated by the upregulation of TTX-sensitive VGSCs during the development of chemotherapy-induced neuropathic pain explains the preventive effect of TTX. A similar mechanism has been shown in other models of neuropathic pain [61]. On the other hand, the discrepancies between the results obtained by TTX in the models of neuropathic pain induced by three different chemotherapeutic agents may be attributable to the different underlying mechanisms by which these anticancer drugs induce neuropathic pain [72].

Other studies evaluated the effects of systemic administration of TTX in preclinical models of neuropathic pain of different etiologies other than those induced by a surgical lesion or chemotherapy. Thus, the subcutaneous administration of TTX suppressed thermal hyperalgesia and mechanical allodynia in a rat full burn wound pain model. Interestingly, the results of this study indicated that TTX reduced thermal hyperalgesia more effectively than morphine, whereas both drugs showed similar effects reducing mechanical allodynia [68]. More recently, the effects of oral administration of TTX pellets have been evaluated in a rat model of postherpetic neuralgia [69]. In this study, intragastric treatment with TTX was able to reduce mechanical allodynia in a similar way than oral pregabalin. In addition, these authors also showed that the intramuscular administration with TTX reduced mechanical allodynia at doses significantly lower than those used during the oral treatments. However, they claimed the TTX pellets described in this article might have a higher therapeutic window than the intramuscular administration of TTX (used in patients). It would be interesting to evaluate this new pharmaceutical form of TTX in more widely used models of neuropathic pain than the unusual model of postherpetic neuralgia induced by resiniferatoxin (RTX). Finally, our group reported that TTX inhibited capsaicin-induced mechanical hypersensitivity in mice [66], which is considered a surrogate model of neuropathic pain [73].

In summary, the systemic or local treatment with TTX has shown efficacy in reducing pain-like behaviors in several animal models of neuropathic pain of different etiology. There is only one study that, by using an adequate systemic dose of TTX, failed to inhibit neuropathic pain in rodents [64]. Therefore, these findings point to the therapeutic usefulness of TTX in neuropathic pain and support the key role that TTX-sensitive VGSCs play in neuropathic pain states (see previous section). Nevertheless, there is still much to learn about the effects of this drug at the preclinical level. In fact, the analgesic effect of TTX in certain preclinical models that mimic highly prevalent painful neuropathies, such as diabetic neuropathy, has never been explored.

### 3.2. Preclinical Studies on Cancer Pain

Chronic cancer-related pain encompasses mixed pathophysiology as a consequence of tissue response to the primary tumor or metastases, and it can be considered a type of mixed nociceptive and neuropathic pain [74]. Although TTX has been used in several clinical trials to relieve cancer-related pain in patients (see next section), it is surprising that this drug is almost unexplored in preclinical models of pain induced by cancer, with only one article. Zhen and collaborators [71] evaluated the role of TTX in bone cancer pain. They found that sustained intrathecal injection of TTX (10 μg/kg, once a day) significantly attenuated mechanical allodynia and thermal hyperalgesia in rats with bone cancer.

With only one preclinical study, it seems clear that more research is needed to evaluate the effects of TTX in models of cancer-related pain. 

## 4. Effects of TTX in Clinical Trials

TTX has been tested in several clinical trials conducted by Wex Pharmaceuticals Inc. to evaluate the response to TTX in patients with cancer-related pain and in patients with neuropathic pain as a consequence of cancer chemotherapy treatment (see Table 3). The clinical development of TTX is more advanced for the indication of cancer-induced pain, since they have already completed two phase III clinical trials with a considerable number of patients (NCT00725114 and NCT00726011). 

### 4.1. Clinical Studies on Cancer Pain

The first clinical trial was a phase IIa open-label, multi-dose efficacy and safety study of intramuscular treatment with TTX in severe cancer pain [75]. In this study, 24 patients were treated in 31 sessions at doses ranging from 15 to 90 µg daily, administered in divided doses, twice a day (b.i.d.) or three times daily (t.i.d.), over four days. The results demonstrated that 17 out of the 31 treatment sessions resulted in clinically significant reductions in pain intensity, with pain relief persisting for up to 2 weeks or longer. The authors concluded that TTX was effective and well-tolerated at doses up to 30 µg b.i.d. [75]. Next, researchers from Wex Pharmaceuticals Inc., further assessed the role of TTX in moderate to severe cancer pain in a larger, multicenter, randomized, double-blind, placebo-controlled trial [76]. Twenty-two centers across Canada were involved in the study and a total of 82 patients were randomized into TTX (N = 41) or placebo (N = 41) groups. In this study, the subcutaneous administration of TTX (30 µg b.i.d.) showed a non-statistically significant trend toward more responders in the TTX arm (42%) vs. placebo arm (31%), based on the primary endpoint (pain intensity difference). However, analysis of secondary endpoints, and an exploratory post hoc analysis, suggested a robust analgesic effect if a composite endpoint is used, including either fall in pain level, or fall in opioid dose, plus improvement in quality of life [76]. At the end of the latter study, all patients were given the opportunity to enroll into a new multicenter open-label study to assess the long-term safety and efficacy of subcutaneous TTX treatment (30 µg b.i.d.) in reducing the intensity of chronic cancer pain. In this new study were recruited 45 patients, of which 47% met the criteria for “responder” (mean reduction in pain intensity of 30% or more from baseline). It was observed that the relief of cancer pain was persistent within successive treatment cycles up to and beyond 1 year, without evidence of tolerance and with an acceptable side effect profile [77].

The promising results of TTX in reducing cancer pain and its good tolerability showed in the clinical trials described above, led Wex Pharmaceutical Inc. to perform a phase III randomized, double-blind, placebo-controlled clinical trial, for the treatment of uncontrolled cancer pain, with a larger number of patients [78]. In this study, 165 patients were recruited, 77 patients in the TTX group and 88 in the placebo group, from 19 centers in Canada, Australia, and New Zealand and the study lasted from 2008 until 2012. The primary analysis showed a statistically significant clinical benefit of TTX over placebo based on the pain endpoint alone (50.8% of patients who received TTX and 34.5% of patients who received placebo were considered responders). Secondary analyses were also very positive in favor of TTX. For example, the mean duration of analgesic effect response of TTX was 56.7 days vs. 9.9 days of the placebo group. In addition, the patient global impression of change was different between TTX and placebo, with most patients who were treated with placebo reporting no change (63.1%) and more than half of the patients who were treated with TTX reporting some degree of improvement (55.4%). Most reported adverse events were generally transient and of a mild to moderate severity [78]. Unfortunately, the number of patients enrolled was considerably smaller than the planned sample size due to associated costs. For this reason, it is considered an underpowered study and the promising results showed by TTX would need to be confirmed through phase III clinical trials with a sufficient number of patients.

In summary, TTX has been evaluated in phase II and phase III clinical trials to verify its safety and efficacy in relieving pain associated with cancer, with very promising results. However, these studies were considered underpowered due to the small number of patients who participated in such studies and, in consequence, further studies with a higher number of patients are needed.

### 4.2. Clinical Studies on Chemotherapy-Induced Neuropathic Pain

TTX has been also tested by Wex Pharmaceutical Inc. for chemotherapy-induced neuropathic pain in a phase II randomized, double-blind, placebo-controlled, multicenter trial (NCT01655823), which has been very recently published [79]. This study was carried out in 125 patients with neuropathic pain as a consequence of anticancer therapy with taxanes or platinum-based compounds to explore safety and efficacy of several doses and dosing intervals of TTX during four consecutive days. The study included 5 dosing cohorts of 25 subjects each (four active and one placebo) and the purpose was to identify a dosing regimen for a future phase III trial, but it was not powered to detect statistical significance between cohorts. The results showed that the two regimens with the maximum dose used in this study (TTX 30 µg once a day or b.i.d) revealed the greatest change from baseline in average weekly pain scores at all time points tested. In addition, TTX 30 µg b.i.d regimen showed the greatest number of responder (subjects with a 30% reduction in pain severity) at any time point tested. Interestingly, TTX displayed a long duration analgesic effect in some patients, a finding that was previously described for this neurotoxin in some cancer pain trials [77,78]. 

As in the previous clinical trials mentioned above, TTX was well tolerated and showed an acceptable safety profile. The authors concluded that according to the results of this study, the development of a new clinical trial for treating chemotherapy-induced neuropathic pain with a higher number of patients is warranted.

## 5. Toxicity of TTX

TTX is a potent neurotoxin, and as such, it can produce severe toxicity that can lead even to death. In fact, TTX is responsible for many human intoxications after ingestion of mainly puffer fish, but also of some molluscs such as mussels, oysters, clams or trumpet shellfishes largely in Asia, although recently, some cases have also been reported in Europe [80]. Symptoms and signs of people intoxicated with TTX include headache, diaphoresis, numbness, nausea, vomiting, abdominal pain, weakness, incoordination, hypotension, cardiac arrhythmias, muscle paralysis, and even death in the most critical cases [81]. In preclinical studies performing to evaluate the capacity of TTX to alleviate chronic pain, no adverse effects such as sedation, respiratory distress or paralysis were observed, at the analgesic doses [57,64,67]. The acute administration of TTX (6 µg s.c.) did not produce motor incoordination in the rotarod test [65]. In addition, the sustained intrathecal administration of TTX (10 μg/kg once a day) did not produce motor alterations in the inclined-plate test [71]. In mice, the reported lethal dose 50 (LD50) of acute TTX administration were 2–10, 12.5–16, and 232 µg/kg for intravenous, subcutaneous, and oral administration, respectively [82]. 

In spite of its well-known potent toxicity, in all clinical trials conducted to date, TTX has shown a very acceptable safety profile. Thus, in a study that evaluated the effects of a single low dose of TTX (5 or 10 µg, intramuscular) on cue-induced drug craving and anxiety in abstinent heroin addicts, none of the participants reported adverse effects during the study [83]. In another clinical trial to evaluate the efficacy of TTX (5 or 10 µg, intramuscular, t.i.d. during 7 days) to alleviate acute heroin withdrawal syndrome, the most common adverse event was irritating pain at the site of injection; however, this side effect was probably not due to TTX as participants in placebo group reported even a higher incidence of such event [84]. The results of the first clinical trial mentioned in the previous section were conducted to evaluate the safety and the efficacy of TTX in cancer-related pain showed that intramuscular TTX administration is safe in a regimen of up to 30 µg b.i.d. for four consecutive days [75]. In this study, and in subsequent clinical trials with patients with cancer-related pain (with different regimens and doses up to 30 µg), the most commonly reported adverse events were transient oral paresthesia and/or oral hypoesthesia, and other mild sensory phenomena. Other reported adverse effects include nausea and dizziness. None of these reported events were considered to pose a safety concern [75,76,77,78]. In the very recent clinical trial evaluating the efficacy of TTX in chemotherapy-induced neuropathic pain (maximum dose 30 µg once a day or b.i.d. for four consecutive days), no serious drug-related safety concerns were reported either. In line with the clinical studies that used similar doses mentioned above, the most frequent drug-related adverse effects were oral paresthesia and oral hypoesthesia [79].

Recently, Wex Pharmaceutical Inc. has published the results of a phase 1 dose escalation study that was performed to evaluate the safety, tolerability, and pharmacokinetics of TTX at clinically relevant exposures in healthy volunteers [85]. In this randomized, double-blind, placebo-controlled study, all safety appraisals show that TTX is safe, well-tolerated, and does not cause cardiac alterations of clinical or regulatory concern or induce any clinically measurable impairment in neuromuscular or respiratory system.

In summary, in all preclinical and clinical studies published to date, therapeutic doses of TTX were well tolerated and showed an acceptable safety profile. In patients, most reported adverse events were generally transient and of a mild to moderate severity, even when TTX was administered over a prolonged period of time. None of the adverse effects described have led to the suspension of clinical trials and, consequently, TTX continues its clinical development.

## 6. Other Natural Toxins Targeting Sodium Channels Tested in Preclinical Models of Neuropathic Pain

There are several naturally occurring venoms targeting voltage-gated sodium channels which have been found in several animals and some plants. These are powerful neurotoxins able to induce not only inhibitory, but also excitatory effects of sodium channels [15]. Some of these inhibitory neurotoxins, in addition to TTX, have been evaluated in preclinical models of neuropathic pain (see Table 4).

Saxitoxin (STX) is a guanidinium shellfish toxin, which similar to TTX, shows potent inhibitory activity of TTX-sensitive VGSC; STX has more than 50 natural analogs which show similar VGSC inhibitory activity to STX, although some of them, such as Zetekitoxin AB, also inhibit TTX-resistant VGSC, including the cardiac isoform Na_V_1.5 [86]. STX or its analogs are practically unexplored in models of neuropathic pain. However, STX has served as the basis for the design of some synthetic derivatives, such as ST2530, a selective very potent inhibitor of Na_V_1.7 isoform which reduced mechanical allodynia induced by the SNI model of neuropathic pain in mice [87].

Another group of toxins present in cone snails are the superfamily of conotoxins (CTX) formed by several families of small peptides with a variety of pharmacological properties. Among CTX families, μ-conotoxins and μO-conotoxins have VGSC inhibitory properties and some compounds have shown preclinical efficacy in reversing mainly inflammatory pain [88]. In addition, the µO-CTX MrVIB from *Conus marmoreus*, which display a good selectivity for Na_V_1.8 isoform, reduced neuropathic pain behaviors induced by nerve injury in rats when administered intrathecally [89].

Spider venoms are also a rich source of VGSC modulators, which are classified into 12 families (NaSpTx1–12), and some of them have demonstrated a good selectivity over VGSC subtypes key for neuropathic pain relief [90], which has led to their evaluation in neuropathic pain models. Thus, intrathecal administration of ProTx-II from the spider *Thrixopelma pruriens*, with a potent and exquisite selectivity for Na_V_1.7, reduced pain behaviors associated to cancer chemotherapy [42] or diabetes [91]. Another spider toxin, named Heteropodatoxin3 (HpTx3), isolated from the venom of *Heteropoda venatoria* also has high potency and selectivity against Na_V_1.7; the systemic administration of HpTx3 reduced mechanical allodynia in the SNI model in mice [92]. HnTX-IV and HwTx-IV isolated from the venom of the spiders *Ornithoctonus hainana* and *Ornithoctonus huwena* respectively, both have good selectivity for several TTX-sensitive VGSC, and they were able to attenuate mechanical allodynia in the SNI model after systemic administration [93,94].

Finally, some peptides found in poisons from certain species of bees, scorpions and sea anemones also target VGSCs, although most of them are activators which produce pain [15].

In summary, although some VGSC inhibitors found in venoms from several animals might be interesting compounds for the development of drugs for neuropathic pain relief, they are still in a very preliminary preclinical research phase and more studies are needed. Therefore, TTX is clearly the neurotoxin targeting VGSC most studied, being the most advanced in its preclinical and clinical development as a drug for neuropathic and cancer-related pain, as mentioned in the previous sections.

**Table 4:** Effects of other toxins targeting sodium channels in preclinical models of neuropathic pain.

Toxin	Organism	Na_V_ Subtype Targeted	Pain Model	Administration	Effect	References
ST2530	Synthetic derived from saxitoxin. Dinoflagellate (*Gonyaulax catenella*)	Na_V_1.7	SNI	systemic	reduced MA	[87]
µO-CTX MrVIB	Cone snail *(Conus marmoreus)*	Na_V_1.8	PNL	intrathecally	reduced MA and TH	[89]
ProTx-II	Spider *(Thrixopelma pruriens)*	Na_V_1.7	CINP (paclitaxel) and PDN	intrathecally	reduced MA and TH	[42,91]
Heteropodatoxin3 (HpTx3)	Spider *(Heteropoda venatoria)*	Na_V_1.7	SNI	systemic	reduced MA	[92]
HnTX-IV	Spider *(Ornithoctonus hainana)*	Na_V_1.2, Na_V_1.3 and Na_V_1.7	SNI	systemic	reduced MA	[93,94]
HwTx-IV	Spider *(Ornithoctonus huwena)*	Na_V_1.7	SNI	systemic	reduced MA	[93,94]

Pain tests (MA: Mechanical allodynia; TH: Thermal hyperalgesia). Pain models (CINP: Chemotherapy-induced neuropathic pain; PDN: painful diabetic neuropathy; PNL: partial nerve ligation; SNI: spared nerve injury).

## 7. Concluding Remarks

Tetrodotoxin has been evaluated for the relief of pain as a consequence of neuropathies or cancer, both at the preclinical and clinical level, showing efficacy and a good safety profile. These studies support the important role that some TTX-sensitive VGSCs, such as Na_V_1.3, Na_V_1.6 and Na_V_1.7, have especially in neuropathic pain. Selective suppression of the abnormal activity generated by changes in these channels in the nervous system, could explain the mechanism of action of TTX in persistent pain relief. Nevertheless, at the preclinical level, there is still much to know about the potential analgesic effects of TTX in these pathologies. In fact, it is striking that the analgesic effect of TTX in cancer pain has been tested in only one study, or that TTX has not been evaluated in certain models of neuropathies as prevalent as diabetic neuropathy. Finally, the promising results obtained by TTX in patients must be confirmed with new appropriately powered clinical trials with a larger number of patients.

## Figures and Tables

**Table 1:** Implication of TTX-sensitive voltage-gated sodium channels in neuropathic pain.

Channel	Normal Localization [16]	Changes of Expression in Pain States
Na_V_1.1	-CNS, PNS-Microglia	-Unclear after PNI in NP [16]
Na_V_1.2	-CNS, very low expression in PNS-SC in lamina I/II	-Unclear after PNI in NP [16]
Na_V_1.3	-Negligible in DRG (embryonic isoform)-SC in lamina I/II	Upregulated in DRG: CCI [21], PDN [22,23], SNI [24,25], SNL [26,27], traumatic nerve injury (human) [28] Upregulate in painful neuromas (human): [29] Trigeminal ganglion: trigeminal neuropathic pain [30,31,32] Upregulated in sciatic nerve: PDN [33] Upregulated in spinal cord: SCI [34]
Na_V_1.4	-Skeletal muscle	
Na_V_1.6	-Nodes of Ranvier-SC-PNS-Epidermal free nerve terminals-keratinocytes-Microglia	Upregulated in DRG: PDN [35], lumbar 5 ventral root transection [36], CINP (oxaliplatin) [37] Upregulated in sciatic nerve: CCI [38] Upregulated trigeminal ganglion: trigeminal neuropathic pain [30]
Na_V_1.7	-PNS in all types of DRG neurons-SC-Epidermal free nerve terminals	Downregulated in DRG: SNL [39], SNI [24,25], traumatic nerve injury (human) [28] Upregulated in DRG: CCI [40], SNI [41], SNL [27], CINP (paclitaxel) [42], cancer-related pain (humans) [42], Herpesvirus quiescent infection [43], painful neuromas (human) [34] Upregulated in spinal cord: CINP (paclitaxel) [42] Upregulated trigeminal ganglion: trigeminal neuropathic pain [44] Upregulated in sciatic nerve: CCI [45]

CINP: chemotherapy-induced neuropathic pain; CNS: central nervous system; DRG: dorsal root ganglia; PNI: peripheral nerve injury; PDN: painful diabetic neuropathy; SCI: Spinal cord injury; SNL: spinal nerve ligation; PNS: peripheral nervous system; SC: spinal cord; CCI: Chronic constriction injury; SNI: spared nerve injury.

**Table 2:** Summary of the effects of TTX on pain studies in laboratory animals.

Administration of TTX	TTX Doses	Effect (None, Moderate, Strong)	Pain Test	Pain Model	Reference
Sciatic nerve blockade	TTX osmotic pump	Strong	MA, TH	SNI and CCI	[61]
Topical DRG	12.5–50 nM/5 µL	Strong (12.5–50 µg)	MA	SNL	[62]
Epidural	25 nM/5 µL	Strong (25 µg)	MA	SNL	[62]
Topical median nerve	Gel pads with TTX	Strong	MA	CCI	[63]
Intraperitoneal	25 nM/5 µL	None	MA	SNL	[62]
	8 µg	None	MA	CINP (vincristine)	[64]
Subcutaneous	1–6 µg	Strong	MA, TH, CA	CINP (paclitaxel)	[65]
	6 µg	Strong	MA	intraplantar capsaicin	[66]
	0.3–6 µg	Strong (1–6 µg)	MA, TH	SNL	[57]
	Acute and subchronic TTX (1–6 µg)	Strong	MA, TH	CCI	[67]
	Acute and subchronic TTX (1–6 µg)	Moderate	MA, TH	CCI-intraorbital nerve	[67]
	8 µg	Strong	MA, TH	burn-induced pain	[68]
Intragastrical	5–20 µg	Strong	MA, TH	Postherpetic Neuralgia (RTX)	[69]
Intramuscular	Acute and subchronic TTX (1–6 µg s.c.)	Strong	MA	Postherpetic Neuralgia (RTX)	[69]
	0.03–1 ug	Moderate	MH	CINP (oxaliplatin)	[70]
Intrathecal	10 µg	Strong	MA, TH	bone cancer pain	[71]

Pain tests (CA: Cold allodynia; MA: Mechanical allodynia; MH: Mechanical hyperalgesia; TH: Thermal hyperalgesia). Pain models (CCI: Chronic constriction injury; CINP: Chemotherapy-induced neuropathic pain; SNI: spared nerve injury; SNL: spinal nerve ligation; RTX: Resiniferatoxin).

**Table 3:** Summary of the analgesic effect of TTX on clinical trials.

Patients	Administration	Doses	Type of Study	Results	Main Adverse Events	References
24	s.c. injections	15–90 μg	Open-label study for severe cancer pain	17 of 31 treatments resulted in clinically meaningful reductions in pain intensity, and relief of pain persisted for up to two weeks or longer	Perioral tingling or other mild sensory phenomena	[75]
82	s.c. injections	30 μg	Placebo-controlled trial for moderate to severe cancer pain	Non-statistically significant trend toward more responders in the TTX arm (42%) vs. placebo arm (31%)	Transient ataxia, mild and related to tingling, numbness, or other transient sensory symptoms	[76]
45	s.c. injections	30 μg	Open-label study for cancer pain	47% met the criteria for “responder”	Mild peri-oral tingling or numbness, transient nausea, irritation	[77]
165	s.c. injections	30 μg	Phase III randomized, double-blind, placebo-controlled clinical trial for moderate to severe cancer pain	Clinical benefit of TTX over placebo based on the pain endpoint alone with a clinically significant estimated effect size of 16.2% (*p* = 0.0460)	Nausea, dizziness, and oral numbness or tingling, generally mild to moderate and transient	[78]
125	s.c. injections	7.5, 15, 30 μg	Phase II randomized, double-blind, placebo controlled trial for chemotherapy-induced neuropathic pain	Changes in pain score were not statistically different between cohorts, due to small trial size and influence of a few robust placebo responders	Mild or moderate oral paresthesia (29.6%) and oral hypoesthesia (24.8%)	[79]
